# Xylitol gummy bear snacks: a school-based randomized clinical trial

**DOI:** 10.1186/1472-6831-8-20

**Published:** 2008-07-25

**Authors:** Kiet A Ly, Christine A Riedy, Peter Milgrom, Marilynn Rothen, Marilyn C Roberts, Lingmei Zhou

**Affiliations:** 1 Northwest/Alaska Center to Reduce Oral Health Disparities, Department of Dental Public Health Sciences, Box 357475, University of Washington, Seattle, WA 98195-7475, USA; 2 Department of Environmental Health & Occupational Sciences, University of Washington, Seattle, WA 98195, USA; 3 Regional Clinical Dental Research Center, University of Washington, Seattle, WA 98195, USA

## Abstract

**Background:**

Habitual consumption of xylitol reduces mutans streptococci (MS) levels but the effect on *Lactobacillus *spp. is less clear. Reduction is dependent on daily dose and frequency of consumption. For xylitol to be successfully used in prevention programs to reduce MS and prevent caries, effective xylitol delivery methods must be identified. This study examines the response of MS, specifically *S. mutans/sobrinus *and *Lactobacillus *spp., levels to xylitol delivered via gummy bears at optimal exposures.

**Methods:**

Children, first to fifth grade (n = 154), from two elementary schools in rural Washington State, USA, were randomized to xylitol 15.6 g/day (X16, n = 53) or 11.7 g/day (X12, n = 49), or maltitol 44.7 g/day (M45, n = 52). Gummy bear snacks were pre-packaged in unit-doses, labeled with ID numbers, and distributed three times/day during school hours. No snacks were sent home. Plaque was sampled at baseline and six weeks and cultured on modified Mitis Salivarius agar for *S. mutans/sobrinus *and Rogosa SL agar for *Lactobacillus *spp. enumeration.

**Results:**

There were no differences in *S. mutans/sobrinus *and *Lactobacillus *spp. levels in plaque between the groups at baseline. At six weeks, log_10 _*S. mutans/sobrinus *levels showed significant reductions for all groups (p = 0.0001): X16 = 1.13 (SD = 1.65); X12 = 0.89 (SD = 1.11); M45 = 0.91 (SD = 1.46). Reductions were not statistically different between groups. Results for *Lactobacillus *spp. were mixed. Group X16 and M45 showed 0.31 (SD = 2.35), and 0.52 (SD = 2.41) log_10 _reductions, respectively, while X12 showed a 0.11 (SD = 2.26) log_10 _increase. These changes were not significant. Post-study discussions with school staff indicated that it is feasible to implement an in-classroom gummy bear snack program. Parents are accepting and children willing to consume gummy bear snacks daily.

**Conclusion:**

Reductions in *S. mutans/sobrinus *levels were observed after six weeks of gummy bear snack consumption containing xylitol at 11.7 or 15.6 g/day or maltitol at 44.7 g/day divided in three exposures. *Lactobacillus *spp. levels were essentially unchanged in all groups. These results suggest that a xylitol gummy bear snack may be an alternative to xylitol chewing gum for dental caries prevention. Positive results with high dose maltitol limit the validity of xylitol findings. A larger clinical trial is needed to confirm the xylitol results.

**Trial registration:**

[ISRCTN63160504]

## Background

Mutans streptococci (MS), more specifically *S. mutans *and *S. sobrinus*, are implicated in the development of dental caries in humans [1]. Xylitol, a naturally occurring sugar alcohol approved for use in food by the U.S. Food and Drug Administration (FDA) since 1963, has been shown to reduce MS levels in plaque and saliva and to markedly reduce tooth decay. Studies involving schoolchildren demonstrated that habitual use decreased dental caries.

A recent review summarized the availability of xylitol-containing products on the internet and in supermarkets and other commercial outlets in the U.S. and included an assessment of their potential to provide a minimally effective dose (6 g) to reduce MS and tooth decay [2]. The review reports that, aside from chewing gum and lozenges, there have been few clinical studies performed on other xylitol-containing products. In all, the existing studies suggested that xylitol can reduce MS levels in saliva and plaque and reduce tooth decay. However, these prospective trials were not designed to assess the relationship between dose or frequency of xylitol use and reductions in MS level or tooth decay. To fill this gap, our group conducted studies to determine the minimal effective dose [3] and frequency [4] of xylitol use when delivered via chewing gum. These studies concluded that xylitol chewing gum dose of 6.9 to 10.3 g divided into at least three uses per day is efficacious in reducing MS, specifically *S. mutans *and *S. sobrinus *(herein referred to as *S. mutans/sobrinus*), level in plaque and saliva. On the other hand, 3.4 g/day or frequencies of use less than 3 times/day were not statistically different from controls even though small reductions were observed.

Xylitol chewing gum and lozenges are widely available and used by consumers in Europe and in Korea, Japan, Thailand, and China. Finland was the first country to implement a national campaign, "Smart Habits" xylitol, to promote xylitol use to reduce tooth decay in children [5]. Similar promotions are occurring in other European and Asian countries especially Japan and Korea where xylitol chewing gum has captured nearly 50% of the chewing gum market. More recently, the U.S. Army implemented the "Look for Xylitol First" initiative to promote xylitol use among deployed troops to improve their oral health [6] and have begun to include xylitol chewing gum in "meals ready to eat" (MRE) rations. Similar programs to address tooth decay in U.S. children have not been adopted in part because chewing gum and hard candies consumption are considered choking hazards and thus not acceptable xylitol delivery vehicles for children [7]. For xylitol to be successfully used in oral health prevention programs for U.S. children, effective means of delivering xylitol in a therapeutic dose and frequency must be identified.

The purpose of this RCT was to test the hypothesis that six weeks of habitual consumption of a xylitol gummy bear snack is effective in reducing *S. mutans/sobrinus *in plaque and to lay the groundwork for a dental caries trial. This study also evaluated the effect of xylitol on *Lactobacillus *spp.

## Methods

### Subjects

Subjects (n = 154) were first to fifth grade children attending Morton and White Pass elementary schools in rural Washington State, USA. The children were initially presented a skit with an oral health message during a general assembly to introduce them to the University of Washington research team. A partnership between the research team and each school was established. The school sent parents an informational letter describing the study and asked permission for their child's participation in the "Gummy Bear Study" (see Additional file 1). Parents interested in having their children participate signed and returned the enclosed consent form along with a brief general health questionnaire (see Additional file 2). Children with reported antibiotic use during the previous two weeks or anticipated its use during the study period were excluded, as were children with a history of gastrointestinal problems. At the first study visit, the assent of each child was obtained prior to study procedures. The University of Washington Institutional Review Board approved the study and related materials.

### Study design

This prospective double-blind, randomized trial employed a three-group design. Children received either 15.6 g (X16) or 11.7 g (X12) xylitol/day, or maltitol (M45) 44.7 g/day. Maltitol was used as the null-comparison because it is only slowly fermentable and a previous study reported no adaptation by MS in plaque to maltitol or xylitol compared to sorbitol and sucrose [8]. Because the gummy bears might be hastily chewed, resulting in small chunks which then would be swallowed thus reducing the xylitol oral bioavailability, higher xylitol doses were used than in previous gum or lozenges studies.

The study design controlled for the frequency and number of gummy bears consumed. Dose of xylitol was varied by combining xylitol and maltitol gummy bears. Pre-packaged and labeled gummy bear unit-doses were distributed in the classroom during school hours three times per day. Gummy bears were not sent home on non-school or missed school days. Subjects were randomly assigned to groups using a computer generated block randomization procedure to ensure a similar proportion of participants in each group. The group assignments were kept by the biostatistician and were decoded at the end of the study. Study and school staff and subjects were blind to group assignment.

### Sample size

The sample size was determined based on intent-to-treat and to provide sufficient power to test the hypothesis that xylitol gummy bears would reduce *S. mutans/sobrinus *and *Lactobacillus *spp. levels in plaque by ≥ 0.75 log_10 _after 6 weeks of exposure. A sample size of n = 41 subjects per group provided 80% power (2-sided, α = 0.05), where a difference in total bacteria counts between pre- and post-intervention was assumed to be 0.75 log_10 _= 5.6-fold reduction. Assuming a loss to follow-up of 10% to 20%, a total recruitment of 51 subjects per group (total n = 153) was necessary to ensure adequate minimum sample size.

### Gummy bears & unit-dose packaging

**Figure 1:**
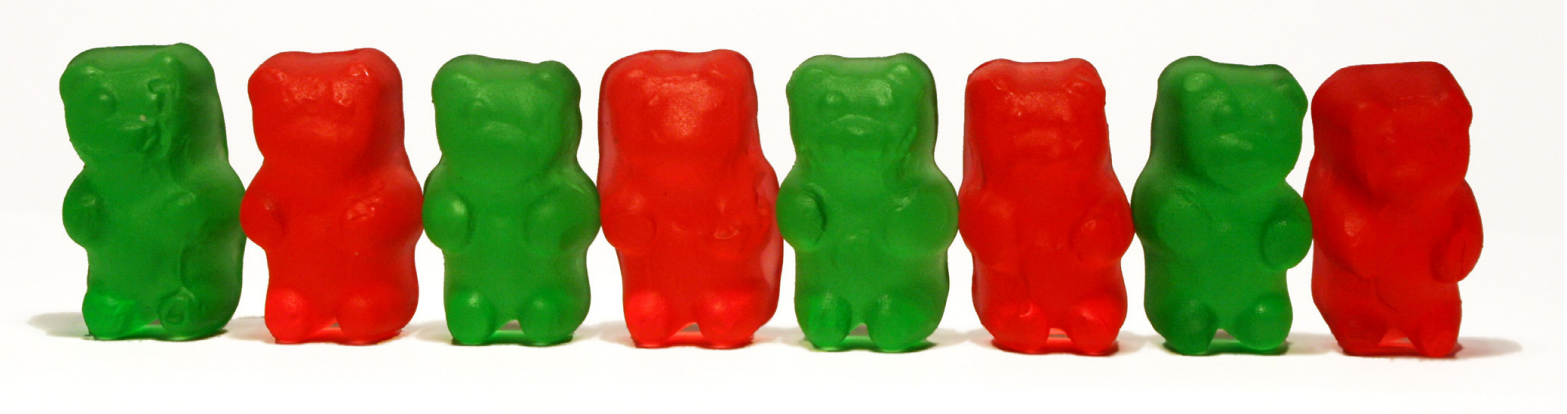
Photograph of Xylitol and Maltitol Gummy Bears.

Both the xylitol and maltitol gummy bear snacks used in this study were produced by Santa Cruz Nutritionals (Santa Cruz, CA) especially for this study and are not available in the general market. The gummy bear snacks looked identical having the same weight (5 g/gummy bear), size, colors (red and green) and flavor (strawberry). They had similar texture and sweetness. Each gummy bear was 30 × 20 × 15 mm in size (see Figure 1). Danisco (Redhill, UK) and Santa Cruz Nutritionals (Santa Cruz, CA, USA) provided the proprietary formulations for the xylitol and maltitol gummy bears, respectively. Each 5 g xylitol gummy bear piece contained 1.3 g (26%) xylitol, 2.7 g (54%) polydextrose, water and gelatin, and minuscule amounts (<1.5%) of citric and lactic acid, mineral oil, carnauba wax and sucralose. Each maltitol gummy bear contained 3.7 g (74%) maltitol, water, gelatin, and similar minuscule amounts of the other ingredients found in xylitol gummy bears. Both types of gummy bears contained similar amounts of substances with laxative effects, i.e. maltitol gummy bear = 3.7 g, xylitol gummy bear = 4.0 g (1.3 g xylitol + 2.69 g polydextrose). However, polydextrose, a non-digestible polysaccharide, has a lower threshold (~90 g/day) for side effects [9] thus the laxative effects may have been slightly higher for the xylitol gummy bear groups.

Study personnel pre-packaged the gummy bears in unit-doses for classroom distribution three times a day: 15.6 g/day = four xylitol gummy bears; 11.7 g/day = three xylitol and one maltitol gummy bears; and maltitol control = four maltitol gummy bears per unit-dose for a total of 12 gummy bears per day for all three groups. Unit-dose packages were labeled with the appropriate randomized ID numbers, date, and period of distribution to facilitate tracking of the administrations each child received per day.

### Gummy bears distribution and adherence procedures

Unit-dose packages were ordered by day and session for each child and pre-packed into bins by classroom before being delivered to the schools weekly. School principals identified people who regularly served as classroom volunteers to hire as community workers for the study. Those women received training in the Responsible Conduct of Research at the University of Washington and were trained to distribute and monitor consumption of the gummy bear snacks in the classroom. The community workers established a schedule with the teachers that allowed them to cover all classrooms three times over the course of the school day at approximately even intervals. The unit-dose packages were distributed according to the child's assigned ID number, date and session number. The community workers observed the consumption of the gummy bears and immediately collected and recorded any unconsumed gummy bears, including those for any absent children. Classroom logs were faxed to the research staff daily to monitor study progress. Incompletely consumed packages of gummy bears were stored in the staff workroom and were picked up by the research staff weekly and checked against the classroom logs for accuracy.

To increase acceptance of the gummy bear snacks and to permit adaptation of the gastro-intestinal system to the polyols, the first week of the study was designed as a ramp-up period. All groups consumed one unit-dose (four gummy bears) corresponding to their group assignment (X16, X12, or M45) on day one and gradually increased to the full three unit-doses (12 gummy bears) by day six. Parents were provided with information on and encouraged to report to the community workers laxative effects common to polyol consumption such as bloating, stomach cramps, flatulence, loose stool, or diarrhea that their child might experience. During the third week of the study, parents were sent a questionnaire on their child's side effects experiences.

### Plaque sampling and culture

Trained research staff collected plaque samples from the children at enrollment (baseline) and six weeks later. Research staff collected samples on-site in the stage area of the assembly hall, from one to four classrooms, throughout the school day. No special instructions were given regarding tooth brushing on collection day. As with our previous xylitol dose and frequency studies, research staff collected samples from the cervical third of the buccal surfaces of all teeth using one sterile Kerr applicator per arch. The samples were placed in 5 mL tubes containing glass beads and 1 mL of pre-reduced saline. The tubes were stored at room temperature and transported the same day from the schools to the laboratory by study staff. Samples were processed the same day or within 24 hours of collection. In the laboratory, plaque was prepared in pre-reduced saline and 10-fold dilutions were prepared separately. For each sample, a modified Mitis Salivarius agar (Difco Laboratories Inc., Detroit, MI, USA) supplemented with 500 μg/mL kanamycin, 1% potassium tellurite solution, and 50 U/mL bacitracin (MSKB) was used to enumerate *S. mutans/sobrinus*. The MSKB medium is more specific than MSB for MS isolation [10,11] but does not distinguish between *S. mutans *and *S. sobrinus *although a previous study found that more than 75% of the isolates were *S. mutans *[11]. Thus, counts from MSKB plates included both *S. mutans *and *S. sobrinus *and is denoted as *S. mutans/sobrinus *in this report to clearly show which species of MS are being referenced. Rogosa SL agar was use to culture and enumerate the *Lactobacillus *spp. [12]. Freshly prepared plates were used. The plaque samples were vortexed to break up the plaque and then diluted. The 10^-0 ^to 10^-3 ^dilutions were plated on MSKB media and incubated in 5% CO_2 _at 36.5°C for five to seven days. The Rogosa SL agar was incubated anaerobically at 36.5°C for seven days prior to enumeration.

### Statistical procedures

Descriptive statistics were used to describe the cohort. Significance of pre- to post-intervention reductions in bacteria levels within groups were assessed using paired T-tests. The analysis of covariance (ANCOVA) was used to assess the association between different treatment groups for pre- and post-intervention bacterial levels. The latter was adjusted for compliance. The SPSS (v.11.5) and SAS (v.9.1) statistical software were used.

## Results

### Baseline

A total of 154 children participated in the three-arm study: 15.6 g xylitol/day (X16, n = 53); 11.7 g xylitol/day (X12, n = 49); and 44.7 g maltitol/day, (M45, n = 52). The mean age (standard deviation) of the children was 8.4 (1.4) years, 55% were boys (85/154) and 90% (138/154) was Caucasian. The mean age and racial/ethnic distribution among the groups were similar (Table 1). At baseline, 42 children (27.3%) did not have measurable *S. mutans/sobrinus *levels (< 10^2 ^cfu/mL. X16 = 17/52, X12 = 12/49, and M45 = 13/53) and 54 children (35.1%) did not have measurable *Lactobacillus *spp. (< 10^2 ^cfu/mL, X16 = 17/52, X12 = 19/49, and M45 = 18/53). Twenty nine (18.8%) children had *S. mutans/sobrinus *levels ≥ 10^4 ^(X16 = 21.1%, X12 = 22.4%, and M45 = 13.2%) and among these, 7 (4.5%) children had levels ≥ 10^6 ^(X16 = 2, X12 = 2, M45 = 3). The mean log_10 _*S. mutans/sobrinus *or *Lactobacillus *spp. levels in plaque between the groups at baseline were not statistically different. Excluding children who did not have measurable *S. mutans/sobrinus *and *Lactobacillus *spp. increased the baseline mean *S. mutans/sobrinus s *and *Lactobacillus *spp. levels, respectively; however, there were still no significant differences in mean reduction between the groups.

**Table 1:** Descriptive statistics and gummy bear compliance by group.

	**Group**			
		
	**Maltitol** ** 44.7 g/day**	**Xylitol ** **11.7 g/day**	**Xylitol ** **15.6 g/day**	**Total**
**N**	53	49	52	154
**Mean Age (SD)**	8.63 (1.4)	8.01 (1.3)	8.60 (1.37)	8.42 (1.38)
**Male (%)**	26 (49%)	31 (63%)	28 (54%)	85 (55%)
**Caucasian (%)**	49 (92%)	42 (86%)	47 (90%)	138 (90%)
**% Missed GB (SD)**	21% (24.7)	25% (28.1)	24% (26.2)	23% (26.3)

### Six week follow-up

At the six week follow-up, 97% of the children completed plaque sampling collection (X16 = 49/52, X12 = 48/49, M45 = 52/53). Overall, children consumed 77% of the total number of gummy bears possible for the study period (X16 = 76.4%, X12 = 73.7%, M45 = 80.4%, Table 1). Nearly all children consumed some gummy bears at each distribution session. However, 15% of the children were frequently absent and consumed less than 50% of their gummy bear allotment while several consumed less than 10% allotted. The proportions of these children were evenly distributed among the groups.

**Figure 2:**
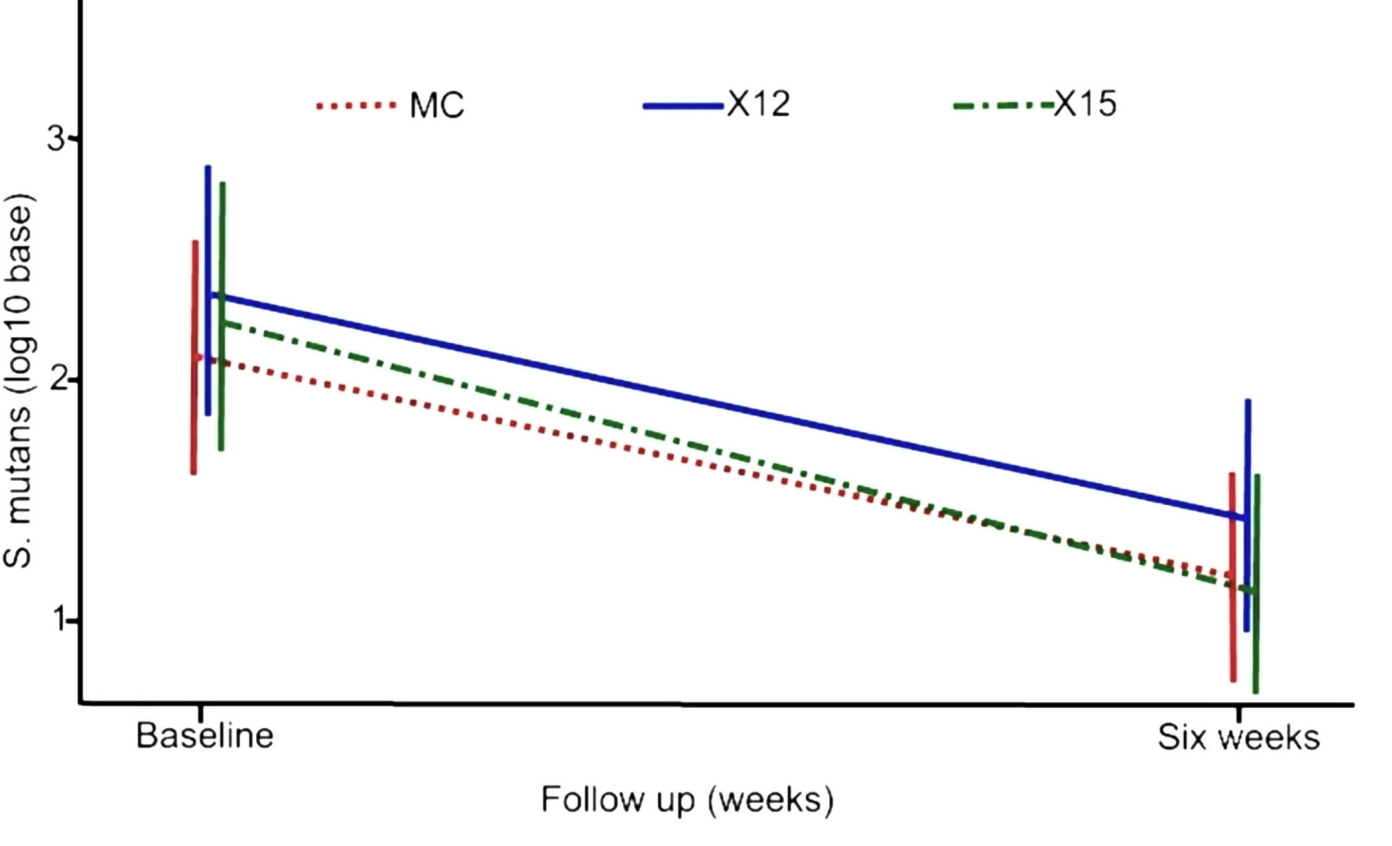
**Mean *S. mutans/sobrinus *levels in plaque at baseline and six weeks of gummy bear exposure**. Mean *S. mutans/sobrinus *levels in plaque at baseline and six weeks for school children exposed to in-school gummy bear snacks containing either xylitol (11.7 g/d or 15.6 g/d) or maltitol three times per day.

The hypothesis tested was whether consumption of xylitol gummy bear snacks at the study dosages over a six-week period, including the one week ramp-up, would reduce *S. mutans/sobrinus *and *Lactobacillus *spp. levels in plaque. A reduction in bacteria levels in response to maltitol exposure was not expected. Results showed significant reductions in *S. mutans/sobrinus *levels pre- to post-intervention for the xylitol groups as well as for the maltitol group (group, log_10 _mean reduction [SD], p-value: X16, 1.13 [1.65], p < 0.0001; X12, 0.89 [1.11], p < 0.0001; and M45, 0.91 [1.46], p < 0.0001, Table 2 & Figure 2). Analysis of the covariance (ANCOVA) showed the reductions in *S. mutans/sobrinus *were not statistically different between the three groups or when xylitol groups (X16 + X12) were combined and compared to the maltitol group (Table 3). Thirty-eight children changed from detectable *S. mutans/sobrinus *to non-detectable levels (X16 = 13/52, X12 = 10/49, and M45 = 15/53). Two children, one from each from group X16 and X12, changed from non-detectable to detectable levels.

**Table 2:** Mean log_10 _*S. mutans/sobrinus *and *Lactobacillus *spp. levels and standard deviation at baseline, six weeks and the differences between six weeks and baseline for schoolchildren exposed to gummy bear snacks containing either xylitol (11.7 g/d or 15.6 g/d) or maltitol.

**Group**	**N**	**Variable**	**Mean**	**Std Dev**	**Paired T-Test**
**M45 (maltitol 44.7 g/d)**	53				
***S. mutans/sobrinus***		Baseline	2.09	1.73	
		Six weeks	1.17	1.55	
		Six weeks – baseline	-0.91	1.46	p < 0.0001
					
***Lactobacillus *spp.**		Baseline	2.11	1.82	
		Six weeks	1.63	1.92	
		Six weeks – baseline	-0.52	2.41	p = 0.13

**X12 (xylitol 11.7 g/d)**	49				
***S. mutans/sobrinus***		Baseline	2.36	1.81	
		Six weeks	1.42	1.64	
		Six weeks – baseline	-0.89	1.11	p < 0.0001
					
***Lactobacillus *spp.**		Baseline	1.79	1.86	
		Six weeks	1.89	1.87	
		Six weeks – baseline	0.11	2.26	p = 0.75

**X16 (xylitol 15.6 g/d)**	52				
***S. mutans/sobrinus***		Baseline	2.25	2.00	
		Six weeks	1.13	1.62	
		Six weeks – baseline	-1.13	1.65	
					
***Lactobacillus *spp.**		Baseline	2.29	1.65	p = 0.75
		Six weeks	2.05	1.91	
		Six weeks – baseline	-0.31	2.35	P = 0.36

**Table 3:** Analysis of the Covariate (ANCOVA) comparing *S. mutans/sobrinus *and *Lactobacillus *spp. levels at six weeks of gummy bear snack exposure between groups*

**Comparison Group**	**F-value**	**P-value**
***S. mutans/sobrinus***		

Maltitol vs. Xylitol groups combined	0.16	0.69
X16 Xylitol vs. X12 Xylitol	0.64	0.43
X16 Xylitol vs. M45 Maltitol	0.00	0.99
X12 Xylitol vs. M45 Maltitol	0.55	0.46

***Lactobacillus *spp.**		

M45 Maltitol vs. Xylitol groups combined	0.13	0.72
X16 Xylitol vs. X12 Xylitol	0.00	0.99
X16 Xylitol vs. M45 Maltitol	0.09	0.76
X12 Xylitol vs. M45 Maltitol	0.10	0.75

*Analysis of the Covariate (ANCOVA) adjusted for treatment assignment, baseline *S. mutans/sobrinus *or *Lactobacillus *spp. level, interaction term between baseline and treatment, and percent gummy bears missed.

Changes in *Lactobacillus *spp. levels pre- to post-intervention were mixed with mean reduction (SD) observed for groups X16 = 0.31 (2.35) log_10_, and M45 = 0.52 (2.41) log_10 _but an increase in levels seen with X12 = 0.11 (2.26) log_10_, (Table 2). However, these mean changes were not significant and were within or near the measurement error, 0.5 log_10_. Adjusting for percent of unconsumed gummy bears did not significantly change the statistical results for either *S. mutans/sobrinus *or *Lactobacillus *spp.

**Figure 3:**
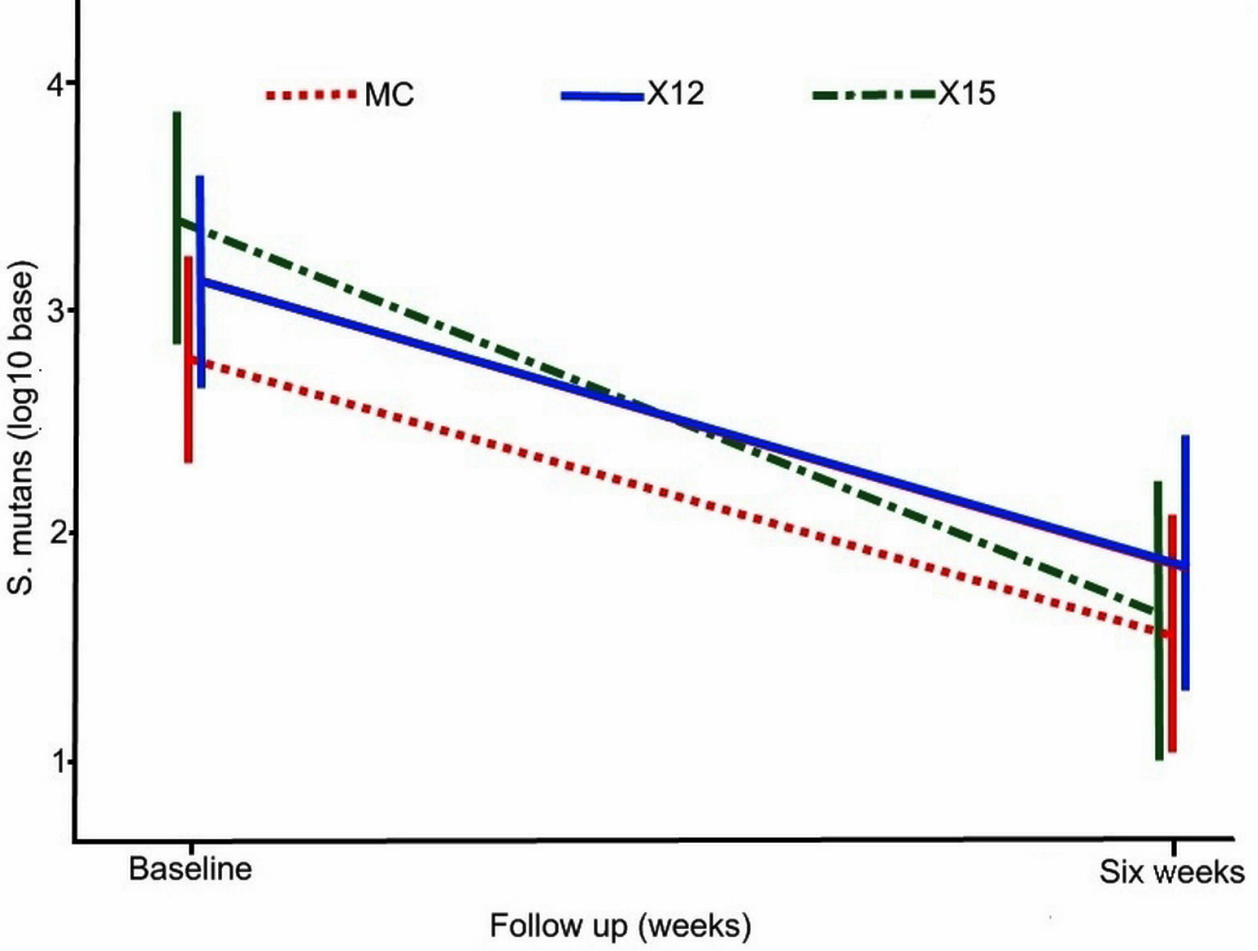
**Mean *S. mutans/sobrinus *levels in plaque at baseline and six weeks excluding children with non-measurable *S. mutans/sobrinus *levels at baseline**. Mean *S. mutans/sobrinus *levels in plaque at baseline and six weeks for school children exposed to in-school gummy bear snacks containing either xylitol (11.7 g/d or 15.6 g/d) or maltitol three times per day after excluding 42 children with non-measurable *S. mutans/sobrinus *levels at baseline.

When children with no measurable *S. mutans/sobrinus *levels at baseline (n = 42) were removed from analyses, the mean log_10 _reduction (SD) for each group increased, X16 = 1.74 (1.67); X12 = 1.21 (1.10); M45 = 1.22 (1.57), and remained significant, P < 0.0001, (Table 4 & Figure 3). Comparison of reductions between the three groups remained non-significant.

**Table 4:** Mean Log_10 _*S. mutans/sobrinus *and *Lactobacillus *spp. levels and standard deviation at baseline, six weeks and the differences between six weeks and baseline after excluding 42 children with non-measurable *S. mutans/sobrinus *levels at baseline for children exposed to xylitol (11.7 g/d or 15.6 g/d) or maltitol (44.7 g/day) gummy bear snack.

**Group**	**N**	**Variable**	**Mean**	**Std Dev**	**Paired T-Test**
**M45 (maltitol 44.7 g/d)**	40				
***S. mutans/sobrinus***		Baseline	2.77	1.44	
		Six weeks	1.56	1.62	
		Six weeks – baseline	-1.22	1.57	p < 0.0001
					
***Lactobacillus *spp.**		Baseline	2.09	1.86	
		Six weeks	1.83	2.03	
		Six weeks – baseline	-0.31	2.40	p = 0.42

**X12 (xylitol 11.7 g/d)**	37				
***S. mutans/sobrinus***		Baseline	3.12	1.39	
		Six weeks	1.87	1.66	
		Six weeks – baseline	-1.21	1.10	p < 0.0001
					
***Lactobacillus *spp.**		Baseline	1.96	1.86	
		Six weeks	1.97	1.90	
		Six weeks – baseline	0.01	2.39	p = 0.99

**X16 (xylitol 15.6 g/d)**	35				
***S. mutans/sobrinus***		Baseline	3.35	1.49	
		Six weeks	1.61	1.75	
		Six weeks – baseline	-1.74	1.67	p < 0.0001
					
***Lactobacillus *spp.**					
		Baseline	2.51	1.85	
		Six weeks	2.41	1.94	
		Six weeks – baseline	-0.14	2.25	p = 0.73

### Snack program acceptability

Post-study informal discussions were held with school principals, teachers, and community workers. All agreed that it was feasible to implement an in-classroom xylitol gummy bear snacks program for caries prevention, and that parents are accepting of the program and children are willing to consume gummy bear snacks daily. Teachers indicated that although challenging initially, it was possible to organize classroom activities to incorporate the snack time, that the community workers were of tremendous value in keeping the program on track and flowing, and that the children were generally well behaved during snack time and followed directions once they became accustomed to the routine. Community workers enjoyed the interaction with the teachers and children and they liked the idea that they were contributing to a program that could improve children's oral health.

## Discussion

This study is part of a series to explore effective xylitol delivery vehicles that can be used in school programs in the U.S. Results from the xylitol dose study [3] showed groups consuming 6.9 g and 10.3 g xylitol per day delivered via chewing gum had significant reductions in *S. mutans/sobrinus *levels in plaque and saliva after five weeks and six months of exposure. The smallest dose, 3.4 g/day, showed a small but not statistically significant reduction. However, the study did not have groups consuming doses between 3.4 g and 6.9 g per day, thus it is possible that doses within this range would also be effective. The xylitol frequency study [4] where the xylitol dose was kept constant (10.3 g/day) and frequency varied (0, 2, 3, and 4), showed a linear response in *S. mutans/sobrinus *reduction with increasing frequency of use. However, a minimum frequency of three administrations per day was required for xylitol chewing gum at therapeutic dose to significantly reduce *S. mutans/sobrinus *levels in plaque and saliva after five weeks of exposure. Twice pay day use group showed a small reduction consistent with the linear line model but was not statistically different from the control. These studies attempted to establish the minimum effective dose and frequency for the use of xylitol. This is important toward establishing guidelines for effective dose and frequency of xylitol use.

Another study in this series assessed the bioavailability of xylitol in saliva over 15 to 20 minutes during and after consumption of different xylitol-containing products including: pellet (2.6 g) and stick (3 g) chewing gums, syrup (2.7 g), and gummy bear (2.6 g) [13]. Participants swallowed normally and at 10 to 11 specified time points, spat into a receptacle until a minimum of 0.5 mL saliva was collected. Xylitol concentration in saliva was measured by high performance liquid chromatography (HPLC). The results showed similar time-curves as well as areas under the curve for the products tested. Thus, similar dose and frequency of xylitol consumption via these products would likely result in comparable *S. mutans/sobrinus *reduction.

In the present study, we tested the effectiveness of gummy bear snacks containing higher levels of xylitol in reducing *S. mutans/sobrinus *and *Lactobacillus *spp. levels in children's plaque after six weeks of exposure. The study was designed and implemented prior to initiation of the bioavailability study when uncertainty remained as to whether different xylitol-containing products would have comparable oral bioavailability. The study design controlled for frequency and number of gummy bears consumed. A school-based randomized controlled clinical trial currently underway is using a lower xylitol dose (7.8 g/day) and a natural fiber (inulin) gummy bear as the control with dental caries as an endpoint.

The results showed that six weeks of habitual xylitol gummy bear consumption reduced the levels of *S. mutans/sobrinus *in plaque compared to baseline levels (Figure 2). A nearly one log_10 _mean reduction of *S. mutans/sobrinus *levels in children is highly significant given that children had lower levels of *S. mutans/sobrinus *infection (mean baseline ~2 log_10_) than among adults in our previous studies (mean baseline ~5 log_10_). The 15.6 g/day group had a slightly greater reduction in *S. mutans/sobrinus *levels but the difference was not significant compared to the 11.7 g/day group. This is in agreement with our dose-response study, which suggested a plateau effect at higher xylitol doses. Controlling for the number of unconsumed gummy bears among the groups did not change the results of within or across group comparisons. This supports findings from previous studies that xylitol reduces MS [14,15]. For a review of these studies, see Maguire & Rugg-Gunn (2003) [16]. More importantly, the results indicate that a gummy bear snack may be an effective method for delivering xylitol. However, these results should be interpreted cautiously given that a significant reduction in *S. mutans/sobrinus *levels was also observed with the maltitol group. Maltitol is a member of the sugar alcohol family that, as a class, is widely considered to be non-cariogenic. However, aside from xylitol, studies involving sugar alcohols, most commonly sorbitol, suggest they have little effect in actively reducing MS levels.

There have been only a few studies on the association between maltitol and MS or dental caries. In a specific pathogen-free (SPF) Sprague-Dawley rats study, Ooshima and colleagues reported that maltitol did not induce dental caries in SPF rats infected with MS including *S. mutans *MT8148R or *S. sobrinus *6715 strain, and replacement of the dietary sucrose content with maltitol resulted in a trend towards caries reduction in SPF rats [17]. However, a study conducted in Estonia found xylitol-maltitol and xylitol-polydextrose candies (49% xylitol in each) were equally effective in reducing MS suggesting that maltitol gave no added benefit to xylitol [18]. In a more recent study comparing xylitol (7.9 g/day) and maltitol (7.1 g/day) chewing gum to control (no gum) consumption over a six month period, Haresaku and colleagues reported that xylitol significantly reduced MS levels in plaque while maltitol increased MS levels [19]. This is clearly incongruous with our findings. However, the authors acknowledged several weaknesses in the study including a non-randomized study design (participants had the option to select their gum flavor which dictated their group assignment), average age of the maltitol group was higher which had a negative association with plaque MS levels, and only 59% of the control group remained in the final analysis. In addition, our study used a very high maltitol dose (44.7 g/day) 4–6 times that of previous studies, thus the results cannot aptly be compared nor can a direct comparison between the 15.6 or 11.7 g/day xylitol dose and the 44.7 g/day maltitol dose be made. Finally, it has been suggested that high or habitual polyols consumption may disturb polysaccharide synthesis leading to plaque and thus MS that are more loosely bound to teeth surfaces, consequently reducing MS levels [20]. Nevertheless, conflicting evidence exists and further research on the association between maltitol, *S. mutans/sobrinus*, and dental caries is needed.

The finding that *S. mutans/sobrinus *reductions were not significantly different between the two xylitol groups was not surprising as our previous dosage study in this series suggested a plateau effect at higher therapeutic doses and our oral bioavailability study showed similar time curves and areas under the curve for xylitol chewing gums, syrup, and gummy bears. The reduction observed with maltitol was also not significantly different from the xylitol groups individually or combined. Although a systematic error in processing, culturing, or enumerating of *S. mutans/sobrinus *in the latter part of the study might explain the lower levels observed in all three groups, it would be unlikely since the reduction observed with the xylitol groups was as expected. Furthermore, sample processing, transport, culturing, and enumeration were carried out by protocol as was done with the baseline samples and the research and laboratory staff had remained unchanged, were highly experienced, and had carried out similar protocols in the previous studies of this series. The mechanical act of frequent chewing of gummy bears may reduce plaque formation and *S. mutans/sobrinus *adhesion but then *Lactobacillus *spp. levels should be similarly affected yet that was not observed. *Lactobacillus *spp. levels remained unchanged. Furthermore, chewing gum studies with sorbitol or sorbitol/maltitol gums have shown no effects on MS plaque or saliva levels [3,21,22]. Nevertheless, the xylitol results would have been strengthened if a true non-actively anticariogenic substance such as sorbitol was used as a control as in previous xylitol studies.

As noted earlier, 27% of the children (X16 = 32%, X12 = 24%, M45 = 25%) did not have measurable *S. mutans/sobrinus *levels at baseline and may have biased the results. Sub-analyses with these children removed showed greater mean log_10 _reductions in *S. mutans/sobrinus *levels for all groups (X16 = 1.74 vs. 1.13, X12 = 1.21 vs. 0.89, M45 = 1.22 vs. 0.91). There were no significant differences between the three groups in the levels of reduction.

This study also assessed *Lactobacillus *spp. response to xylitol exposure as the literature contains conflicting information. Juric and colleagues reported a reduction in these bacteria after two months of xylitol chewing gum exposure [23]. The Turku sugar studies [24] and the Belize xylitol study [21] with much longer follow-up periods also reported reductions in salivary *Lactobacillus *spp. On the other hand, in a study comparing the effects of chewing xylitol (5 g/day), sorbitol, and fructose gum over a four week period on *S. mutans *and Lactobacilli in plaque, Loesche and colleagues reported only xylitol significantly reduced *S. mutans *levels and there was no effect on the Lactobacilli levels for any gum [14]. Similarly, a recent study among young adults (21 to 24 years of age) reported no change in salivary *Lactobacillus *spp. in response to xylitol chewing gum consumption three times per day (6 g total) over a three week period but reported a significant reduction in salivary MS level [25]. The current study found that neither of the xylitol groups nor the maltitol group had an effect on *Lactobacillus *spp. level. It is possible that xylitol selectively affects and reduces *S. mutans/sobrinus *levels without altering *Lactobacillus *spp. levels, the other bacteria implicated in development of caries. Alternatively, the six weeks follow-up period of this study might not have been of sufficient length to detect the effects on *Lactobacillus *spp. Nevertheless, xylitol studies with dental caries as an end-point have repeatedly shown xylitol's effectiveness in caries reduction [26].

Finally, the post-study discussion with school principals, teachers, and community workers suggested that it is feasible to implement a school-based xylitol gummy bear snacks program. Teachers were willing and able to incorporate the three times per day snack program into their classroom curriculum with minimal disruption after a brief adjustment period. Parents were willing to allow their children to participate in an oral health program and to consume gummy bear snacks at school to reduce tooth decay. Children were willing to eat most of the gummy bears provided at each distribution. A significant degree of commitment, cooperation, motivation, and effort from all parties involved was necessary to successfully implement and carry out the program. The information and experience gained from the current study were valuable in strategizing and developing the protocols used to implement and carry out the xylitol gummy bear snacks randomized clinical trial being conducted among kindergarten children in an elementary school district in Cleveland, Ohio. A lower xylitol dose is being used.

This study had several limitations. Plaque collection was not standardized by plaque weight but rather by sampling the arches of teeth. This may be viewed as qualitative and may affect the quantitative analysis. However, the effect is minimized by standardization of sampling technique, by staff training, and by the use of staff with previous experience in this sampling technique. Furthermore, our previous studies with xylitol chewing gums showed that *S. mutans/sobrinus *levels in plaque sampling at screening were comparable to those at baseline for subjects and throughout the study among controls. The use of the manufacturer's (Santa Cruz Nutritionals) high dose maltitol (44.7 g) formula rather than the sorbitol/polydextrose formula was necessary because the sorbitol formula would not congeal properly to form the gummy bear during production. The reduction observed with high dose maltitol may have weakened our xylitol results. The inherent limitations of the setting and its capacity to deliver a public health intervention are an additional weakness. In this study, the intervention was delivered in a school setting and was subject to school closures and early dismissals, children's absences and cooperation, and classroom activities. For example, we experienced fluctuating cooperation of the children (e.g., some wanted to negotiate the number of bears to be consumed on some days, some preferred a specific color). Despite these difficulties, the local community workers who delivered the gummy bears made valiant attempts at getting the gummy bears to the children. Overall, 73% of the children consumed at least 75% of the total possible gummy bears distributed.

Based on our findings, it is feasible to develop and implement xylitol-based caries preventive programs in structured settings such as schools and daycare. These structured environments offer an opportunity to achieve xylitol frequency and dose compliance. However, implementing a new activity into an institutional setting would undoubtedly be a challenging task and require the acceptance and commitment of all parties within the setting (e.g., school administration, teachers, parents and students, etc.).

## Conclusion

Xylitol gummy bear snack consumption at therapeutic dose and frequency reduced *S. mutans/sobrinus *but not *Lactobacillus *spp. levels in plaque after six weeks of habitual exposure. Xylitol doses in the high therapeutic range such as those in this study, 11.7 g and 15.6 g/day, produced similar reductions confirming a plateau of effect previously reported in this series of studies. High dose maltitol (44.7 g/day) also reduced *S. mutans/sobrinus *levels in plaque after six weeks of exposure. It is feasible to implement a xylitol gummy bear snack program in the school setting or daycare. Further study is needed to confirm the results and help determine if implementing such a program should be seriously considered to reduce the burden of dental caries among U.S. children, particularly those in high-risk populations.

## Competing interests

The authors declare that they have no competing interests.

## Authors' contributions

KAL was the project director and PM was the principal investigator for the overall series of xylitol studies. CAR coordinated this study. MR contributed to the design of this study, supervised and carried out the study procedures and contributed in the editing of the manuscript. MCR was the director of the laboratory that processed the studies' samples, generated all the numbers for *S. mutans/sobrinus *and *Lactobacillus *spp., and contributed to the studies' laboratory design and procedures. LZ was the data manager and analyst. All authors read and approved the final manuscript.

## Pre-publication history

The pre-publication history for this paper can be accessed here:



## Supplementary Material

Additional file 1 **Gummy bear study informational school letter to parents**. Letters sent to parent prior to recruitment with information about the study. Click here for file

Additional file 2 **Gummy bear study baseline health questionnaire**. Questionnaire used to obtain descriptive and general health information. Click here for file
