# Supplementary material for: Xylitol gummy bear snacks: a school-based randomized clinical trial
Source: BMC Oral Health. 2008 Jul 25;8:20. doi: 10.1186/1472-6831-8-20 (PMC2527560; doi:10.1186/1472-6831-8-20)
Supplement: Additional file 1 — Gummy bear study informational school letter to parents. Letters sent to parent prior to recruitment with information about the study. [file 1472-6831-8-20-S1.pdf]

Appendix I

Gummy bear study informational school letter to parents

Dear Parent,

Our school is participating in a school-based dental project with researchers at the University of Washington. Researchers at the UW have been studying xylitol, a natural sweetener and food additive approved by the FDA that prevents tooth decay. They are developing xylitol containing snack foods for use as healthy snacks in school settings to help eliminate cavities.

The American Academy of Pediatric Dentistry and Dental Associations throughout the world recommend xylitol to help reduce cavities. Many foods and sweet snacks, candies, and gums currently sold in the supermarket contain xylitol but not in amounts large enough to provide protection against cavities. The researchers at the UW have completed studies in adults to determine how much xylitol is needed each day and how often to decrease the amount of cavity causing bacteria on teeth. The purpose of this project is to see if xylitol containing gummy bears will decrease the amount of cavity causing bacteria in children's mouths. Some children who participate in the study will receive gummy bears that contain maltitol instead of xylitol so the results can be compared with the test results from children who receive xylitol. Maltitol, like xylitol, is a natural, sugar-free, sweetener that is a food additive approved by the FDA.

The researchers are asking for your permission to allow your child to participate in this project. Please check below if you do or do not give permission. If you agree to allow your child to participate, please read and sign the Consent Form attached and complete the Health Questionnaire. This questionnaire asks about your child's medical health. Before the study is started, your child will be read an assent form explaining the study. He/she may choose not to participate in the study.

If you are interested in having your child participate, but have some questions that you would like answered before giving permission for your child to participate, please contact the study team at the toll free number: 1-866-XYLITOL (1-866-995-4865). Please keep in mind that participation is entirely voluntary. You are free to accept or refuse participation and your child's participation and rights to all other regular school activities will not be affected by your decision.

Thank you for your consideration.

Please check one of the choices below. Please return this letter, consent form, and the Health Questionnaire to the school by April 11, 2007.

☐ NO, I do NOT give permission. (You need only return this form)

☐ YES, I give permission for my child to participate in the study.
(Please also read and sign the Consent Form and complete the Health Questionnaire. The second copy of the consent form is for you to keep.)

If you give permission, please fill in your name and phone number in order that the study team may contact you if they need clarification regarding any information that you provide about your child's health.

Parent's Name:______________________________Phone number:________________

3-5-07
