# Supplementary material for: Xylitol gummy bear snacks: a school-based randomized clinical trial
Source: BMC Oral Health. 2008 Jul 25;8:20. doi: 10.1186/1472-6831-8-20 (PMC2527560; doi:10.1186/1472-6831-8-20)
Supplement: Additional file 2 — Gummy bear study baseline health questionnaire. Questionnaire used to obtain descriptive and general health information. [file 1472-6831-8-20-S2.pdf]

Appendix II

UNIVERSITY OF WASHINGTON
GUMMY BEAR STUDY

BASELINE HEALTH QUESTIONNAIRE

For Study Staff use only
Study ID#: _________
☐ Consent Form Signed

Date: ______________________________

If you have given your permission for your child to participate in the Xylitol Gummy Bear study and signed the consent form, please complete this questionnaire. It asks you about your child's medical health. This questionnaire will take about 5 minutes to complete. All answers are for our records only and will be kept confidential. If you are not comfortable answering a question you may skip it.

<u>Questions about you and your child's background:</u>

Q1. Is your child who is participating in the study ☐ male or ☐ female?

Q2. What is your child's Birth Date: Month______Day_________Year________

Q3. Do you consider your family as Hispanic or Latino? ☐ Yes ☐ No

Q4. Do you consider your family as:

☐ White ☐ African American or Black

☐ Asian ☐ Native Hawaiian/Pacific Islanders

☐ American Indian or Alaska Native

(continue on the next page)

Date: ______________________ Study ID: __________________

The next set of questions asks about your child's medical health. Please answer the questions as best you can. Again, you do not have to answer any question that you are not comfortable answering.

Q5. Do you consider your child's health as:

☐ Excellent ☐ Very Good

☐ Good ☐ Fair ☐ Poor

Q6. Is your son/daughter being treated for any condition by a physician now?

☐ YES→ Please explain. ____

____

☐ NO→*Go to next question.*

Q7. Is your child currently taking or has he/she taken any medications in the past month? This includes prescription and over the counter medications.

☐ YES→

Name	Dose	Frequency

☐ NO→*Go to next question.*

Q8. Does your child have allergies to any medicine or food or to latex?

☐ YES→Please list. ____

☐ NO→*Go to next question.*

(continue on the next page)

Date: ______________________ Study ID: __________________

Does your child have any of the following medical conditions? Please mark "Yes" or "No".

Q9.	Ever had injury to face, jaws, neck	☐ YES	☐ NO	Q15.	Have Sinusitis	☐ YES	☐ NO
Q10.	Ever had Temporomandibular joint disease (TMJ)	☐ YES	☐ NO	Q16.	Have Bronchitis	☐ YES	☐ NO
Q11.	Have *(now)* acid-reflux/heartburn	☐ YES	☐ NO	Q17.	Have Asthma	☐ YES	☐ NO
Q12.	Have Gastritis	☐ YES	☐ NO	Q18.	Have Pneumonia	☐ YES	☐ NO
Q13.	Have Irritable bowel syndrome	☐ YES	☐ NO	Q19.	Have Diabetes	☐ YES	☐ NO
Q14.	Have Sjogren's syndrome	☐ YES	☐ NO	Q20.	Have Phenylketonuria	☐ YES	☐ NO
		Q21. Have behavioral condition **If YES, do NOT indicate which** (*e.g. Attention Deficit, psychiatric illness, depression, anxiety/panic attacks, etc.*)				☐ YES	☐ NO

Thank you for taking the time to complete this questionnaire. It is possible that your child will be screened out of this study. Your child can choose not to take part in this study. If you have questions or concerns at anytime during the study, please feel free to contact the study staff.
